# FIGGsolver: An interactive algorithm that guides the genealogy process in forensic investigative genetic genealogy^☆^^☆☆^

**DOI:** 10.1016/j.fsisyn.2026.100720

**Published:** 2026-10-30

**Authors:** Lawrence M. Wein, Malo Sommers

**Affiliations:** a Graduate School of Business, Stanford University, Stanford, CA 94305, United States of America; b Institute of Computational and Mathematical Engineering, Stanford University, Stanford, CA 94305, United States of America

**Keywords:** Forensic investigative genetic genealogy, Probabilistic analysis, Stochastic dynamic programming, Family trees

## Abstract

The genealogy process is a key bottleneck within forensic investigative genetic genealogy, which is a novel approach to solving violent crimes and identifying human remains. Building on our earlier work, we develop FIGGsolver: an interactive algorithm that guides the genealogist through the tree-building process by specifying precise actions for the genealogist to take at each point in time. The algorithm has been used to solve actual cases and plans are underway to make FIGGsolver widely available to the forensic investigative genetic genealogy community. We describe the algorithm in non-mathematical terms and collect insights gathered from its construction and use.

## Highlights

• We describe FIGGsolver, an algorithm that guides the genealogist in the FIGG process.
• It has been used to solve actual cases and will soon be available to the FIGG community.
• Insights are provided from the construction and use of FIGGsolver.

## 1 Introduction

### 1.1 Background

Forensic Investigative Genetic Genealogy (FIGG) is a novel approach to solving cold criminal cases and identifying unidentified human remains [1]; the approach can also be used by adoptees to find their biological parents. Genotyped DNA samples from a crime scene are submitted to a third-party service (e.g., GEDmatch Pro or FamilyTreeDNA) that generates a list of biological relatives (called *matches*) to the unknown criminal (called the *unsub*, an abbreviation for unidentified subject). Genealogists then construct a family tree using these matches, with the aim of identifying the unsub. If the genealogists believe that they have identified the unsub, then law enforcement undertakes confirmatory DNA testing. The genealogy process is a key bottleneck (both in terms of time to solve and likelihood of solving) in solving cases by FIGG, and the genealogy process has become more cumbersome due to recent changes at Ancestry.com [2], lending a greater urgency to the efficient construction of family trees.

A mathematical model of the genealogy process was constructed and analyzed in [3], which resulted in a proposed strategy that attempts to identify the unsub (we used the term *target* instead of unsub in [3]) as quickly as possible. This strategy determined the next action for a genealogist (e.g., which match to investigate and find its ancestors, or which ancestors to descend from and find their descendants). In [3], the strategy was tested on randomly generated variants of 17 cases from the DNA Doe Project, and the simulation results suggested that the strategy could solve cases much more quickly than a simple-minded benchmark strategy.

Building on the analysis in [3], we have created FIGGsolver, which is an interactive algorithm that specifies the precise actions for the genealogist to perform. The genealogist still carries out the genealogy using, e.g., birth, death and marriage records.

### 1.2 Enhancements to [3]

FIGGsolver is an improvement on the algorithm in [3] in several major ways. Whereas the model in [3] works with randomly simulated cases and makes several optimistic informational assumptions regarding the relationship between two matches and the relationship between a match and the unsub, FIGGsolver infers the relationship between two matches and the relationship between a match and the unsub solely from the cM values, which tells us the total amount of DNA shared between two individuals (either two matches or a match and the unsub), and (if a match is successfully identified) the date of birth. In addition, both the ascending actions and the descending actions have been completely revamped. Rather than ascending a match in isolation, we now perform a *community ascent* (see Section 5.1 for a description), which involves ascending a primary match in parallel with other matches that are related to the primary match. Rather than descending from a single set of ancestral couples of some matches, we now perform a dual descent from two sets of ancestral couples, and a corresponding new definition of the benefit of a dual descent allows us to set the ultimate goal of finding the unsub’s parents (as opposed to any intersection or connecting couple found during a descent, as in [3]).

FIGGsolver also incorporates an override option, where genealogists can choose which matches to investigate. While this option allows genealogists to avoid the possible overreliance on algorithmic recommendations, its main purpose is to allow genealogists to pre-populate the model with portions of an existing family tree when they want to use FIGGsolver on stalled cases rather than de novo cases. Finally, while we do not recommend reference sampling (i.e., when law enforcement requests a DNA sample from a person in a partially constructed family tree) as a general rule (see [4] for a study suggesting that its benefits may be modest), FIGGsolver uses the results in §6 of Supporting Information of [4] to determine the most desirable person to test if the genealogist concludes that a reference test is necessary.

On a related note, FIGGsolver allows genealogists to augment a case by adding new matches (e.g., reference tests, or new matches becoming available on a third-party service) to a case that has already been worked on. The only tricky part is updating the clustering, which is discussed briefly in Section 4.1.

### 1.3 Goal of the paper

In contrast to [3], we do not present the detailed mathematics of the algorithm, which precludes reproducibility or the assessment of mathematical correctness. Nor do we provide any computer code. However, plans are underway to make FIGGsolver widely available to the FIGG community.

FIGGsolver has been used to solve actual (rather than simulated) cases that had resisted solution (some details from over a dozen cases – most involving Black or Hispanic unsubs, which are typically more difficult to solve due to more distant genetic relatives and sparser genealogical records – were presented at [5]). However, we offer no empirical validation of solved cases, case-based evidence or comparative performance data.

In summary, this paper provides neither a detailed mathematical description of FIGGsolver nor an empirical validation of its performance on actual cases. Rather, our goal is two-fold: to provide a non-mathematical overview of the algorithm to improve its transparency, and to summarize the main insights that can be gleaned from our approach, thereby providing a structured way to understand and tackle this complex and important problem.

### 1.4 Limitations of FIGGsolver

Readers and potential users should view FIGGsolver merely as a promising algorithm: it is not a panacea, and cannot completely overcome the challenges that plague FIGG, such as misattributed parentage, adoption, incomplete or inaccurate genealogical records, database representation bias, and endogamy. Also, FIGGsolver uses the estimates in [3] of the identification probabilities of matches, a person’s parents, and a person’s child, based on 17 DNA Doe Project cases that were not chosen randomly. Of most concern, the current version of FIGGsolver does not exploit information about geographical location, surnames or bioancestry, which genealogists often use to help solve FIGG problems. Consequently, as noted in [3], FIGGsolver is intended to aid – and not replace – the genealogist, which is partly why we added the override option mentioned in Section 1.2. Therefore, genealogists should not over-rely on FIGGsolver and should not necessarily stop working on a case if the algorithm fails.

### 1.5 Relevant literature

We now attempt to position FIGGsolver within the existing literature on FIGG. The basic approach of ascending from matches and looking for most recent common ancestors, and descending from ancestors and looking for connecting couples, has been central to FIGG since its inception, and predate the development of our algorithm; e.g., see the books [6], [7] that were published prior to the seminal Golden State Killer case [8], as well as several insightful FIGG case studies (e.g., [9], [10]) that elucidate this approach. Indeed, the problem formulation in [3] explicitly adopts this approach.

Moreover, specialized tools have been developed to aid genealogists in various aspects of the FIGG process, including the relationship between two people based on their shared cM [11], [12], clustering tools based on the autosomal matrix [13], [14], [15], tree-building tools such as AutoTree [16], and related tools such as What Are The Odds (WATO) [17], which computes the conditional probability for possible tree locations of the unsub. We use [11], [12] directly as a pre-processing step (see Section 4.2), and – while inspired by the non-hierarchical clustering in [13] – use a hierarchical clustering approach that is necessary for our purposes as another pre-processing step (see Section 4.1). We note that these specialized and highly valuable tools essentially solve static statistics problems, whereas we are attempting to solve a much more difficult sequential decision problem. Finally, although not central to our study, we refer readers elsewhere for a discussion of the legal, ethical, privacy and governance issues related to FIGG (e.g., [1], [18], [19], [20], [21], [22], [23], [24], [25], [26]).

### 1.6 Outline of the paper

The outline of the paper is as follows. The mathematical problem that we are trying to solve is described in Section 2, and a broad overview of our approach is provided in Section 3. Two key pre-processing steps are described in Section 4, and ascending and descending actions are described in Section 5 and Section 6, respectively. In Section 7, we outline a number of modifications made to the algorithm to improve its robustness, and we collect insights from constructing and using FIGGsolver in Section 8.

## 2 The problem to solve

This section contains a description of the mathematical problem that we are trying to solve.

### 2.1 Initial data

FIGGsolver requires three types of initial data, beginning with the estimated year of birth of the unsub. For each match (e.g., from GEDMatch or FamilyTreeDNA), we also need the total cM shared between the match and the unsub. Finally, the algorithm requires the total cM shared between every pair of matches, which is typically contained in the autosomal matrix (sometimes called the A-matrix).

### 2.2 Decisions

At each step of the process, FIGGsolver proposes one of three actions: ascend (more precisely, perform a community ascent, as described in Section 5.1) from a match, dual-descend from a pair of lists that contain potential ancestral couples of the unsub (as in [3], we assume that it is always possible to find a spouse of someone in the family tree, and so we deal with ancestral couples – abbreviated here by ACs – rather than individual ancestors; see Fig. 1), or stop (if it is deemed that the case is unlikely to be solved, which in our experience occurs very rarely).

**Fig. 1:**
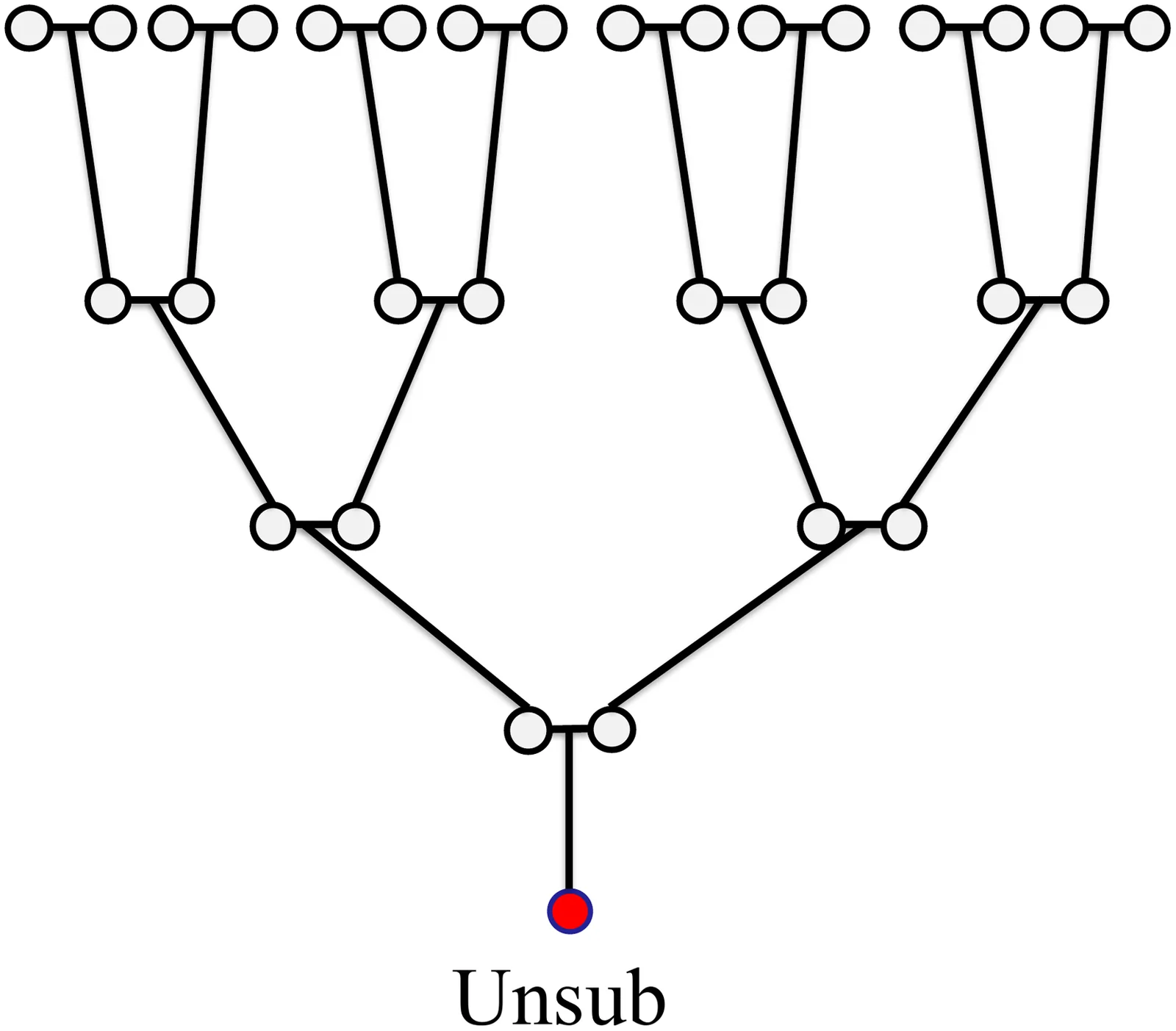
The unsub’s ACs include one set of parents, two sets of grandparents, four sets of great-grandparents, and eight sets of great-great-grandparents.

### 2.3 Interactive information flow

When the proposed action is to ascend from a match (community ascent involves ascending the primary match and its close relatives in parallel, and its description is deferred until Section 5.1), FIGGsolver asks the genealogist a yes/no question of whether they can identify the match, which means determining the identity of the match based on the match’s name and email address. If the genealogist cannot identify the match, then the current action is completed and FIGGsolver computes the next action. If the genealogist can identify the match, FIGGsolver asks the genealogist for the match’s year of birth. After the genealogist enters the match’s year of birth, FIGGsolver tells the genealogist to ascend from this match for a specified number of generations, and to enter the ACs of the match that are found in the highest generation. For example, if the algorithm asks the genealogist to ascend three generations from match 6, there are four sets of great-grandparents that are specified by 6:FFP, 6:FMP, 6:MFP and 6:MMP, where F represents father, M represents mother and P represents parents, and, e.g., 6:FMP is match 6’s father’s mother’s parents. After the genealogist selects the set of ACs that they found, the genealogist is asked whether any of these ACs were already identified as ACs of previously investigated matches, and if so, to describe these equivalencies (e.g., 6:FFP is the same couple as 4:FMFP); such ACs are referred to as *duplicate ACs*, and are typically the most recent common ancestor (MRCA) couple between two matches. This completes the ascending action.

When the action is to dual-descend from two lists (continuing the example, a list may include 6:FFP and 6:MFP if the genealogist found two of the four sets of great-grandparents of match 6), FIGGsolver specifies how many generations to descend from each of the two lists (e.g., the dual descent might consist of descending two generations from one list and one generation from the other list), and then asks a yes/no question of whether the genealogist found a *connecting couple* (e.g., marriage, but more specifically two people who have a child together) between a descendant from each of the two lists (although it is possible that a connecting couple consists of a descendant of one of the two lists and a descendant from a list that was descended in a previous descent), which completes the dual-descend action.

It is worth highlighting the distinction between a duplicate AC and a connecting couple (called an intersection in [3]). A duplicate AC is found during an ascent and is typically an MRCA couple between two matches (e.g., ascending the green and pink matches, who are first cousins, by two generations each leads to their common set of grandparents in the first step in Fig. 2), whereas a connecting couple is found during a descent and consists of two individuals who, e.g., are married or have at least one child together (e.g., descending the green AC by two generations and the purple AC by one generation leads to the red connecting couple in Fig. 3).

**Fig. 2:**
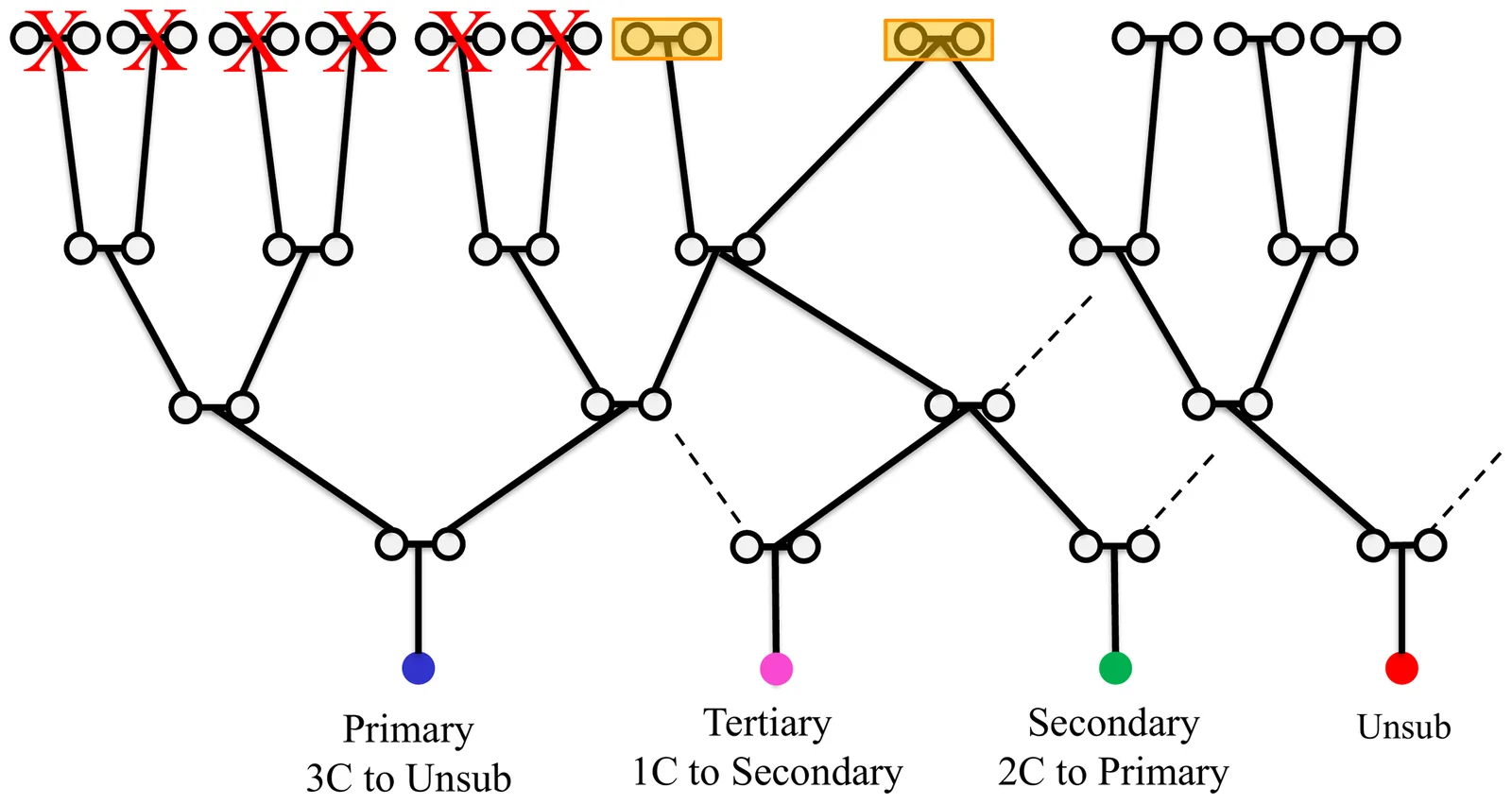
An example of a community ascent performed in three steps: (1) ascend the tertiary match and the secondary match for two generations each and find their MRCA couple, (2) ascend this MRCA couple by one generation and ascend the primary match by three generations and find the MRCA couple between the primary match and the secondary match, and (3) ascend this second MRCA couple by one generation, thereby reducing the number of possible ACs that could be the MRCA couple between the primary match and the unsub from eight to two.

**Fig. 3:**
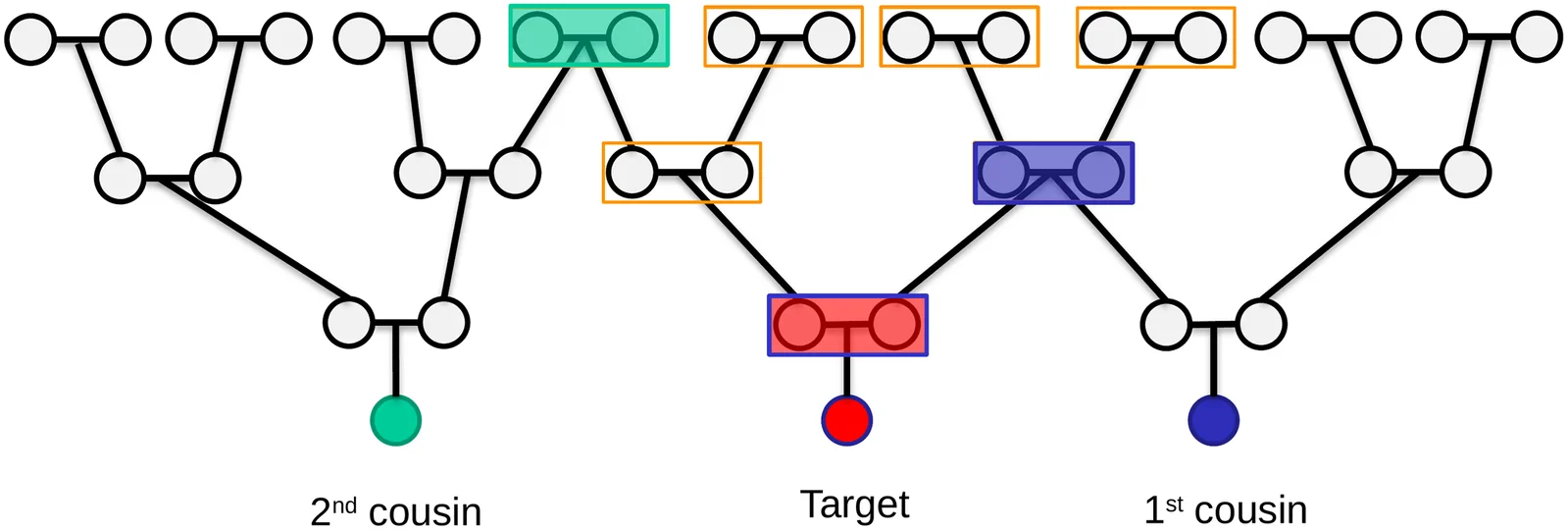
An example of a dual descent. Descending the great-grandparents (green) in the unsub’s paternal side by two generations and descending the maternal grandparents (purple) by one generation leads to the discovery of a connecting couple, which is the unsub’s parents (red).

### 2.4 Objective

The decisions are made with the aim of finding the unsub’s parents with as small a family tree as possible. If the unsub’s parents are found, it is up to the genealogist and law enforcement to determine which child of the unsub’s parents is the unsub; in the adoption setting, the adoptee is the unsub and the unsub’s parents are the adoptee’s biological parents.

**Fig. 4:**
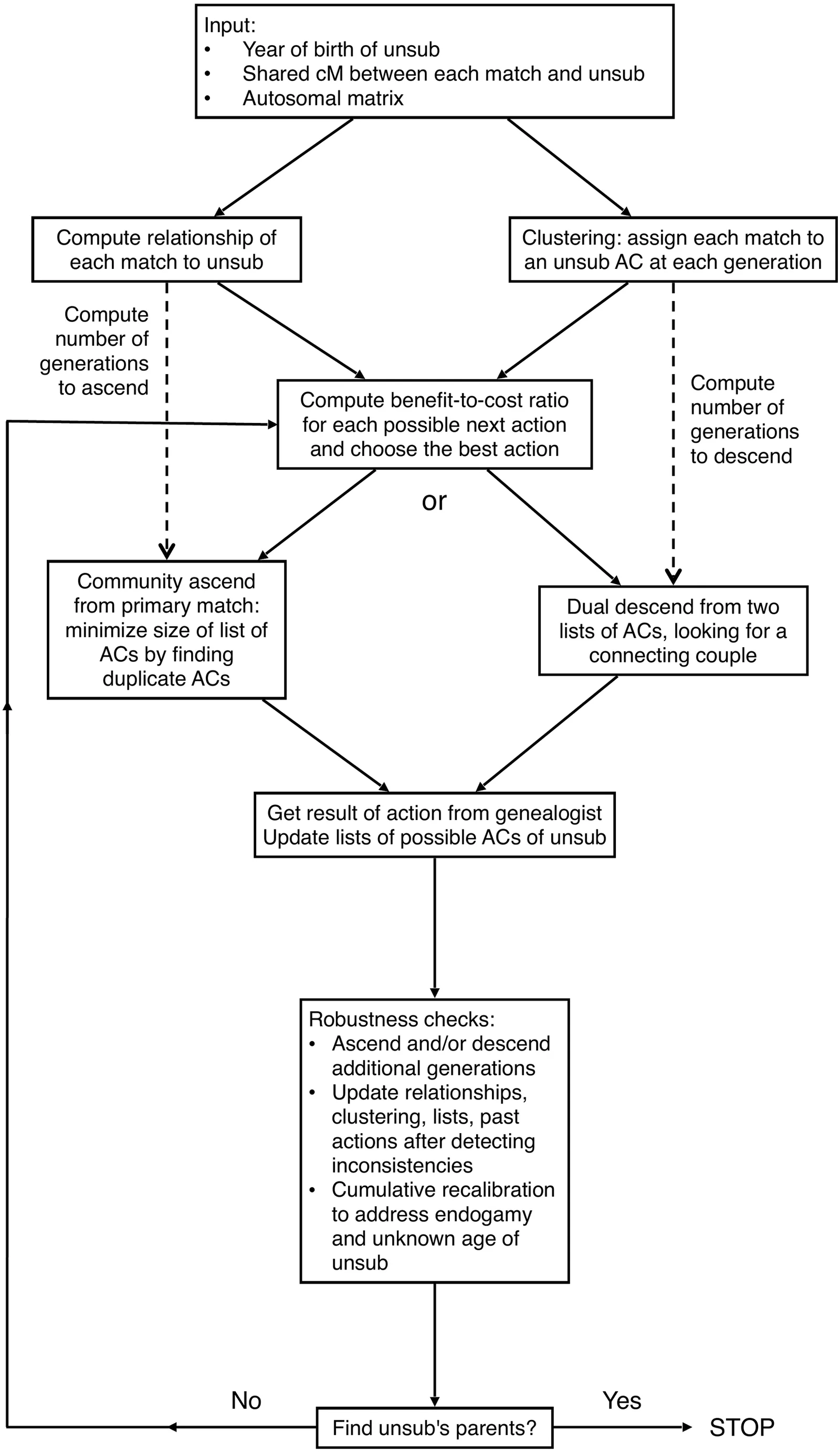
An Overview of FIGGsolver.

## 3 The big picture

An overview of FIGGsolver is depicted in Fig. 4. Our problem falls under the umbrella of stochastic dynamic programming, which is a technique to model and analyze multi-stage decision problems under uncertainty [27]. The reader might keep in mind a similar, but far more complex, problem of controlling a self-driving car. In that setting, one keeps track of the state of the system, which would describe the environment (e.g., pedestrians, other vehicles, traffic lights, weather conditions), and at each point in time makes decisions (accelerate/decelerate, steering). After each decision is made, the system state is updated (incorporating not only the most recent actions but also any random events, such as the arrival of new cars and pedestrians, that have recently occurred). At each point in time, decisions are made to achieve a goal (e.g., safely and legally travel from point A to point B in as little time as possible).

To describe the state of the system in our problem, note that prior to beginning our investigation, all we know is that the unsub has a collection of unknown ACs, consisting of one set of parents, two sets of grandparents, four sets of great-grandparents, eight sets of great-great-grandparents, and so forth (Fig. 1). As the genealogist populates the family tree associated with our investigation, for each specific AC of the unsub, we maintain a list of couples who might be the true AC of the unsub. We determine which couples go into which lists based on the autosomal matrix and the amount of shared cM each match has with the unsub, as explained later. These lists – one for each AC of the unsub – of couples constitute the most important part of the state of our system (other information also has to be maintained, such as whether we previously descended from each list, and which ACs were in the list when we previously descended from it).

As described in Section 2.2, our decision at each point in time is a community ascent (ascend a specific set of matches for a specific number of generations), a dual descent (descend from a pair of lists of ACs for a specific number of generations), or stop the investigation. As noted in Section 2.4, our goal is to find the unsub’s parents with as small a family tree as possible. Due to the huge dimension of the state description and large number of possible actions, our stochastic dynamic program suffers from the “curse of dimensionality,” which precludes finding the optimal solution to this problem using conventional techniques [27].

Instead, we take a somewhat myopic approach. When we are in the current state, FIGGsolver computes a benefit-to-cost ratio for each of the thousands of possible actions that could be taken, and tells the genealogist to take the action that has the largest benefit-to-cost ratio. Our problem is one of finding a needle in a haystack, where the needle is the unsub’s parents, and the haystack consists of all possible couples who could be the unsub’s parents, as inferred by the current family tree. In our calculations, the benefit of an action is the expected reduction in the amount of hay (i.e., how many couples can be excluded from being the unsub’s parents, and hence discarded from the haystack, due to finding a duplicate AC and/or a connecting couple), and the cost is the number of new people we add to the family tree. As in [3], our myopic approach looks ahead for only one action if the action is a dual descent, and looks ahead through the next dual descent when the action is a community ascent (i.e., there may be a number of community ascents to perform before it is beneficial to perform a dual descent).

As in [3], we compute the benefit and cost by computing the *transition probabilities* in the stochastic dynamic program [27]; i.e., for every possible current state and every possible current action, what is the probability that we end up in any given state after taking the action. The most difficult part of these calculations is computing the probability that any current list of ACs contains the true AC of the unsub.

Before describing community ascents and dual descents in more detail, we describe two key pre-processing steps.

## 4 Two pre-processing steps

This section contains a description of two important pre-processing steps: hierarchical clustering of matches using the autosomal matrix, and computing the relationship of a match to the unsub. Due to the modular nature of these two steps with respect to the overall algorithm, if more sophisticated approaches (e.g., based on all the SNP data rather than on only the total cM) are developed, they can be incorporated into the algorithm.

### 4.1 Hierarchical clustering of matches using the autosomal matrix

In [3], we assumed a perfect AutoCluster tool (e.g., as if the tool in [13], [14] was perfectly accurate) that correctly assigns each match to exactly one AC in each generation above the unsub. Here, we use hierarchical clustering (see §12.4.2 of [28] for details) based on the autosomal matrix to perform this task. More specifically, all matches are related to the unsub and hence are related to the unsub’s parents. Each match is believed to be related to one of the unsub’s two sets of grandparents, and are clustered into two groups, one for each set of grandparents. Until we start building the family tree, we do not know which group represents the unsub’s maternal side of the family tree and which represents the paternal side, but the clustering algorithm can nonetheless construct the two groups. Suppose we have 1000 matches and we assign 600 of them to one grandparent cluster and 400 to the other grandparent cluster. Every match is believed to be related to one of the unsub’s four sets of great-grandparents, and the hierarchical clustering algorithm will take the 600 in the first cluster (at the grandparent level) and split them into two clusters at the great-grandparent level (e.g., 350 in the first cluster and 250 in the second cluster), and split the 400 in the second grandparent cluster into two clusters at the great-grandparent level (e.g., 250 in the third cluster and 150 in the fourth cluster), and so on into higher generations. The hierarchical nature of the clustering enforces consistency in the clustering decisions, so that, e.g., if a match is related to the unsub’s mother’s mother’s parents, then it must also be related to the unsub’s mother’s parents (Fig. 5).

The clustering results are used for two purposes. First, it is useful information when deciding which match to investigate next. For example, if we already found an AC that we believe to be an MRCA couple between the match and the unsub, then there may be no need to investigate other matches that are in the same cluster at the generation of this MRCA couple. Second, we can use the hierarchical clustering results to compute the generation of a connecting couple in a dual descent (see Section 6).

**Fig. 5:**
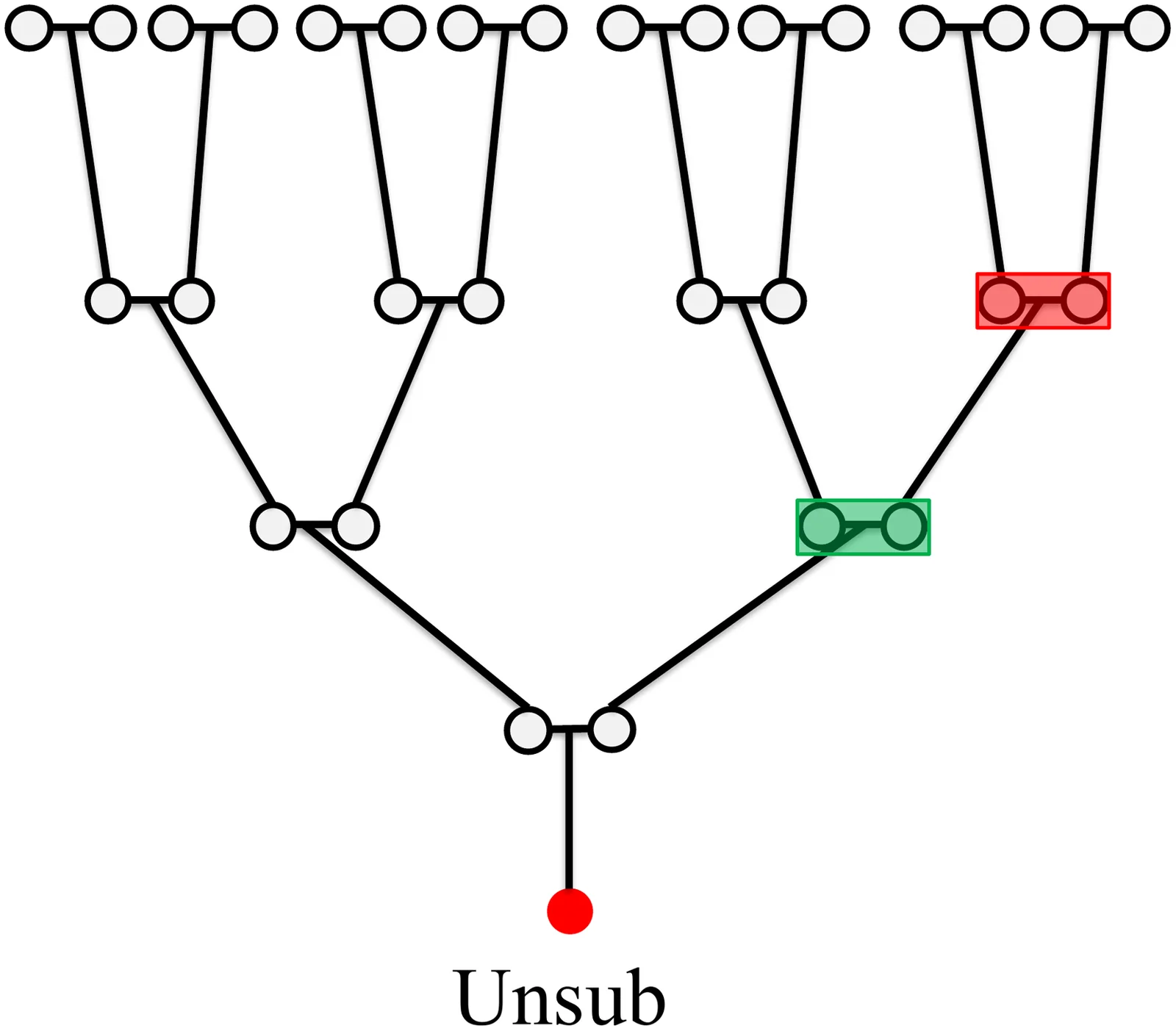
Hierarchical clustering enforces consistency across generations, so that, e.g., if a match is related to the unsub’s mother’s mother’s parents (red AC of the unsub), then it must also be related to the unsub’s mother’s parents (green AC of the unsub).

Finally, the accuracy of the hierarchical clustering results varies by generation and by match, and in Section 7 we calculate which of the clustering results are reliable, and modify the benefit-to-cost ratios of ascents and descents accordingly.

The description above applies to new cases. An augmented case occurs when new matches are added to a case that has already been worked on (but not solved). If there are old matches that do not appear in the new matrix, we maintain their existence in the new matrix, and assume they share zero cM with any new match. Because the clustering results impact the lists that provide the foundation of all past decisions and hence of the current family tree, we do not attempt a full re-clustering of all old and new matches. Rather, all old matches retain the original clustering, the new matches are added to the autosomal matrix, and each new match is clustered based on its shared cM value with the old matches and with all other new matches that have higher shared cM values with the unsub than the new match.

### 4.2 Computing the relationship of a match to the unsub

We use equation (4) in the Supporting Information of [3], along with results in [11], [12], to compute the probability distribution of the relationship between each match and the unsub, based on the shared cM between the match and the unsub. These computations are required in the computation of the benefit-to-cost ratio for each possible ascending action, and hence are performed prior to investigating (and possibly identifying) a match. Therefore, the calculations do not incorporate the relative ages of the match and the unsub. However, after we investigate and successfully identify a match, we have knowledge of the match’s year of birth, and we use the ages of the match and the unsub to update the probability distribution of the relationship between the match and the unsub. For example, the amount of shared cM between a person’s grandparent, aunt/uncle, half-sibling, niece/nephew, and grandchild are all very similar, and the difference in age between the unsub and the match can narrow down the set of likely relationships and refine the probability distribution. This refined probability distribution is used to compute the number of generations to ascend the match to get to the generation where the MRCA couple between the match and the unsub resides, which is a key instruction to provide to a genealogist during a community ascent.

## 5 Ascent

We describe community ascents in Section 5.1, and consider investigating other relatives of the primary match in Section 5.2.

### 5.1 Community ascent

The analysis in [3] assumes that each match is ascended in isolation, and if a match has already been investigated, and if a different match is a closer relative to the previously-investigated match than to the unsub, then there is no need to investigate this uninvestigated match. However, the decreasing fertility rate of women over the last several centuries [29] causes us to want to reduce the size of the list of ACs prior to descending the list, which in turn lowers the number of people added to the family tree when seeking a connecting couple via a dual descent. In this subsection, we introduce the concept of *community ascent*, which attempts to achieve this goal.

More specifically, when FIGGsolver chooses an ascending action, it focuses on a single match, which we call the primary match. However, when investigating the primary match, we also investigate in parallel all *secondary* matches that are more closely related to the primary match than the primary match is to the unsub, all *tertiary* matches that are more closely related to a secondary match than the secondary match is to the primary match, and all *quaternary* matches that are more closely related to a tertiary match than the tertiary match is to its corresponding secondary match. Prior to identification of a match, we assess the closeness of a relationship between two people by their shared cM (i.e., a higher shared CM implies a closer relationship), and after identification we assess it by the generation of the MRCA couple between these two people (i.e., a lower generation of the MRCA couple implies a closer relationship). Hence, it is possible that, e.g., after identifying a secondary match and updating its relationship with the primary match, we decide not to ascend the secondary match because the generation of the MRCA couple between the secondary and primary matches is higher than the generation of the MRCA couple between the primary match and the unsub. Note that siblings, children, grandchildren, nieces, nephews, grandnieces and grandnephews of a match offer no new information for us, and so these close relatives are not investigated.

After computing the various MRCA generations related to all primary, secondary, tertiary and quaternary matches that could be successfully identified, we perform the community ascent in multiple dual-ascent steps, working backward starting with dual ascents of quaternary and tertiary matches, followed by dual ascents of tertiary and secondary matches, followed by dual ascents of secondary and primary matches, followed by a solo ascent of the primary match. At each dual ascent, we are looking for a MRCA couple between two matches, and continue to ascend only from these couples. Fig. 2 depicts an example of a three-step community ascent that reduces the size of the list of great-great-grandparents of the primary match from eight to two.

The benefit-to-cost ratio is refined to reflect community ascents by increasing this ratio for the investigation of each possible primary match by two factors: one related to the number of supporting (secondary plus tertiary plus quaternary) matches that a primary match possesses, and the other related to the expected size of the reduction in the list size as a result of the community ascent.

### 5.2 Investigating other relatives of the primary match

In Section 5.1, we investigated secondary matches for which the generation of the MRCA couple between the secondary match and the primary match is less than or equal to the generation of the MRCA couple between the primary match and the unsub. Nonetheless, if the list of ACs of the primary match in the MRCA generation of the primary match and the unsub is not sufficiently small, it may still be worthwhile investigating secondary matches for which the generation of the MRCA between the secondary match and the primary match is larger than the generation of the MRCA between the primary match and the unsub. Hence, before moving on to the next action, we compute the benefit-to-cost ratio of these other secondary matches, prioritizing secondary matches that have lower generations of the MRCA between the secondary match and the primary match.

If we do investigate one of these secondary matches, we implement it by first ascending the secondary match to its MRCA generation with the primary match, also investigating in parallel the secondary match’s tertiary or quaternary matches that have yet to be investigated. The goal of investigating the secondary match is to find a duplicate AC among the ACs of the primary match in the generation of the MRCA couple between the primary match and the unsub; this duplicate AC will be a true AC of the unsub. After ascending the secondary match, this duplicate AC can be sought by either descending the secondary match’s ACs from the MRCA generation of the primary match and the secondary match down to the MRCA generation of the primary match and the unsub (in this case, one of the descendants will be one-half (e.g., husband or wife) of an AC in the list of ACs of the primary match in the MRCA generation of the primary match and the unsub), or by ascending the list of ACs of the primary match in the MRCA generation of the primary match and the unsub up to the MRCA generation of the primary match and the secondary match. We compute the number of people added to the family tree in each of these two approaches, and then choose the approach that adds fewer people (which typically – but not always – is to ascend). In either case, if we are successful in finding the duplicate AC, we can identify the true AC of the unsub, and hence reduce the corresponding list to a single correct AC.

After assessing all of the uninvestigated secondary matches in this way, we then compute the benefit-to-cost ratio of investigating any other matches that have a shared cM of at least 30 with the primary match (30 cM is chosen as a rough threshold of an interesting relative to primary match i), prioritized by larger shared cM values. We perform the ascents as in the previous paragraph. These matches are typically only investigated in difficult cases where primary matches have no secondary matches. An example is provided in Fig. 6.

**Fig. 6:**
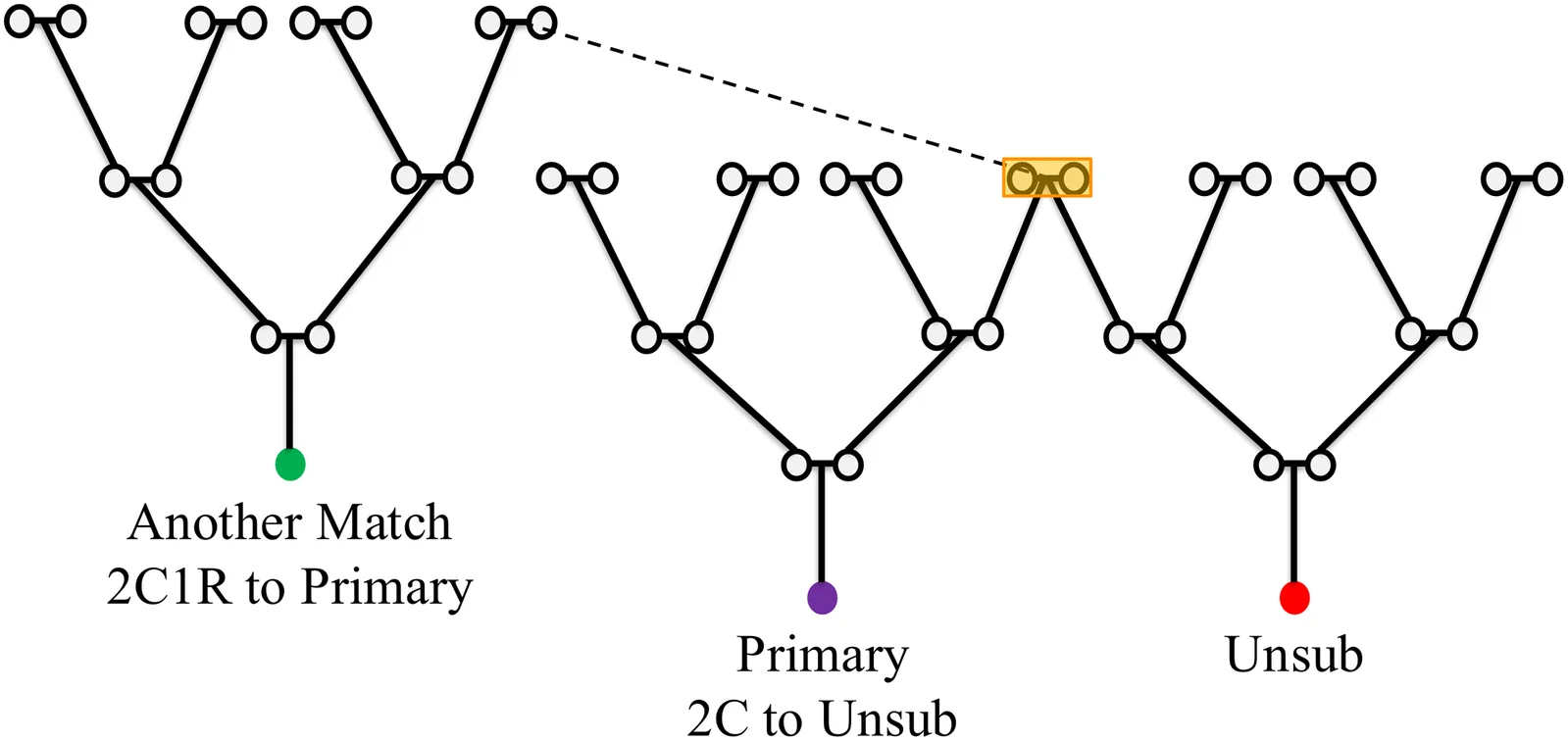
An example of investigating other relatives of the primary match. Ascending the primary match leads to a list of four possible ACs that are the true MRCA (yellow) between the primary match and the unsub. Another match, which is a 2C to the primary match’s mother (i.e., a 2C1R to the primary match), is ascended 3 generations to the generation of the MRCA between it and the primary match. We then either descend this other match’s ACs by one generation or ascend the primary match’s ACs by one generation (typically the latter is less work), and find the dashed line, which allows us to find the true MRCA between the primary match and the unsub.

## 6 Descent

The descent analysis has been entirely revamped relative to [3]. We describe the new goal in Section 6.1, introduce a new benefit metric and analyze dual descents in Section 6.2, and consider partial dual descents in Section 6.3.

### 6.1 A new goal: Finding the unsub’s parents

The goal of a dual descent is to find a *connecting couple*, which is when a descendant from one AC and a descendant from a different AC have at least one child together. By construction, the unsub is a descendant of the connecting couple, and hence all ancestors of the connecting couple (as well as other descendants of these ancestors) can be ignored (i.e., they are now obsolete in terms of helping us find the unsub’s parents). If a connecting couple is in generation 1 (as in Fig. 3), then it is the unsub’s parents, and we are done. However, it is possible for the connecting couple to be in a higher generation, in which case it will be on one side of the unsub’s family tree. For example, if we had a second-cousin match to the unsub whose MRCA couple with the unsub is the unsub’s father’s father’s parents and a different second-cousin match with the unsub whose MRCA couple with the unsub is the unsub’s father’s mother’s parents, then a dual-descent from the unsub’s father’s father’s parents and from the unsub’s father’s mother’s parents would lead to a connecting couple that was the unsub’s father’s parents, which implies that the unsub is a grandchild of the connecting couple.

Note that in [3], we treated the detection of any connecting couple as a success that ends the search process. Hence, we found at most one connecting couple. In reality, the lower the generation that the connecting couple resides in, the more valuable the connecting couple. Here, we generalize our algorithm by allowing the benefit of detecting a connecting couple to depend on what generation it resides in, and by continuing the search until we find the unsub’s parents.

### 6.2 Dual descent and a new benefit metric

At each point in time, we compute the cost and benefit of all possible dual descents, and find the one with the highest benefit-to-cost ratio, which is compared to the highest benefit-to-cost ratio of all possible ascents in order to choose the next action. A dual descent descends from one list of ACs in a particular generation and another list of ACs in a possibly different generation. We first descend from the list in the higher generation until we get to the generation where the lower list resides, and then we descend from both lists together until we get to the generation of the connecting couple in the dual descent, which can be computed using the hierarchical clustering results. More specifically, the location of each of the two lists can be expressed as a binary tree (not to be confused with the family tree) where the “child” in the tree is an ancestral couple in the generation below, and the generation of the connecting couple is the highest generation in which each of the two binary trees has the same child. For example, if the first list corresponds to the unsub’s father’s father’s mother’s parents, and the second list corresponds to the unsub’s father’s mother’s mother’s parents, then the connecting couple would reside in generation 2.

As in [3], the cost of a dual descent is the number of new people added to the family tree. Our new metric for the benefit of finding a new connecting couple is the expected reduction in the number of descendants that could possibly be the unsub’s parents, which is the reduction in the total number of descendants in generation 1 of the ACs in all of the lists if we find the connecting couple, times the probability of finding the connecting couple.

FIGGsolver typically uses an incremental approach to find the unsub’s parents: e.g., it may first find a connecting couple in generation 4, and then perform a dual descent that consists of finding the connecting couple’s children and descending from a different list of ACs that reside in generation 4 or higher, ideally resulting in a new connecting couple in generation 3. This process is repeated and the next dual descent looks for a connecting couple in generation 2, and the process is repeated once more to find the unsub’s parents.

### 6.3 Partial dual descent

It is possible for a dual descent to involve descending zero generations from one of the two lists, which we call a partial dual descent. In this case, we descend one (higher-generation) list and hope to find a duplicate in the other (lower-generation) list. Successful partial dual descents involve people who are biologically related to each other, which leads us to implement two refinements in this subsection.

First, because the shared cM is a much more direct and accurate measure of the relationship between any pair of matches than the clustering results, we disallow a partial dual descent if it appears that none of the matches that generated one list are related to any of the matches that generated the other list.

The second refinement for partial dual descents is that – as in Section 5.2 – it may be more workload-efficient if we ascend from the lower-generation list rather than descend from the higher-generation list. We compare the workload of each approach and choose the approach with the smaller workload.

## 7 Improving robustness

In this section, we enhance the robustness of the algorithm by addressing the uncertainty in the relationships (Section 7.1) and in the clustering (Section 7.2).

### 7.1 Addressing the uncertainty in relationships

Even after updating the probability distribution of the relationship of a match to the unsub after learning the match’s year of birth, there is often considerable uncertainty in the relationship, which translates into uncertainty about the number of generations to ascend the match to reach the generation of the MRCA couple between the match and the unsub. Thus far, we have made decisions based on the most likely relationship, and here we hedge against this uncertainty.

**Continue Ascending Secondary Matches:** When we dual-ascend a secondary match and a primary match, if we do not find a duplicate AC after ascending the secondary match to the generation of the MRCA between the secondary and primary match, we continue ascending each match one generation at a time until we find a duplicate AC or until we ascend the primary match to the generation of the MRCA between the primary match and the unsub.

**Assess an Additional Generation of Ascent After a Failed Dual Ascent:** We assess (i.e., compute the benefit-to-cost ratio and then perform the action if it exceeds the threshold in [3]) whether to dual-ascend an additional generation from a primary and secondary match if the dual ascent fails to find a duplicate AC. If there is more than one secondary match, we perform a separate assessment for a dual ascent of the primary match and each secondary match. Assessing this decision after a failed dual ascent would be cumbersome for two reasons: it would require an additional back-and-forth with the genealogist, where the genealogist would have to provide the list of all ACs found (in every generation) during the dual ascent. Instead, we assess this decision prior to the dual ascent by computing the conditional probability that we find a duplicate AC if we dual-ascend an additional generation given that we do not find a duplicate AC in the original dual ascent.

**Assess an Ascend–descend Action After a Failed Dual Descent:** If a dual descent fails to find a connecting couple, it may be because we underestimated the number of generations to ascend the original primary match to get to the MRCA generation of the primary match and the unsub. Hence, after a failed dual descent, we assess (i.e., compute the benefit-to-cost ratio and then perform the action if it exceeds the threshold in [3]) ascending an additional generation from one of the two lists of ACs that were involved in the dual descent, and then performing a single (as opposed to dual) descent from these new ACs (i.e., from the parents of the ACs in the original list). For example, if the dual descent in Fig. 3 fails to find a connecting couple, then we would assess the ascend–descend action of ascending the green AC by one generation, and then descend the green AC’s two sets of parents by three generations. We compute the benefit-to-cost ratio for the ascend–descend action from each of these two lists in isolation; if both ratios are above the threshold, we carry out the ascend–descend action with the larger ratio and then carry out the other action only if the first ascend–descend action is unsuccessful.

**Impose Age Constraints During Descents:** Using 15 years of age as the minimum age for giving birth, we impose age constraints to ensure that descents continue to – but not beyond – the targeted generation. For example, if the unsub was born in 1950 and we are trying to descend to generation 2 (i.e., the generation of the unsub’s grandparents), then after every new female descendant (or female partner of every new male descendant) is discovered, we stop descending from this descendant if they were born after 1920. After descending to generation 2, we descend from any female descendant for an additional generation if she was born before 1905.

**Perform Updates After Detecting Inconsistencies:** Here, we embark on a more ambitious and comprehensive undertaking to hedge against uncertainties. This approach uses information that we gain from the genealogist (beyond the ages and the ACs — in particular, inconsistencies in any duplicate ACs or connecting couples that we encounter in the family tree) to update our system state (i.e., the lists of the unsub’s ACs), our clustering results and even our past actions. In this way, as we dynamically construct the family tree, we detect (via duplicate ACs and connecting couples) and fix inconsistencies to improve our estimates about relationships and clustering. These improvements also help to compensate for endogamy and for an inaccurate estimate of the year of birth of the unsub. For ease of presentation, for the remainder of this subsection, we refer to connecting couples as also being duplicates because their inconsistencies are handled in a similar manner.

A duplicate couple can be detected during any community ascent or dual descent, and the first encounter with this couple may have occurred during a community ascent or dual descent. In addition, during a community ascent, we ask the genealogist to identify duplicate couples in any generation, not just the generation of the MRCA couple between the match and the unsub. An inconsistency arises if the two versions of the duplicate couple are found in different generations or even in different clusters within the same generation. For example, we might dual ascend two matches by two generations each, expecting to find a duplicate AC, but we find the duplicate AC after ascending the first match by two generations and the second match by one generation.

When we find an inconsistency, we carry out the following sequence of six steps (some of which are skipped in some instances):

• *Consolidate:* The two versions of the duplicate couple are consolidated into one couple.
• *Recompute relationships:* A duplicate couple generates mathematical constraints that must be satisfied by the relationships (to the unsub) of the matches involved in the inconsistency. We keep a running track of all known duplicate couples, and whenever we detect a new duplicate couple, we add new constraints to a mathematical optimization problem that chooses the relationship to the unsub of each match so as to maximize the joint probability (these probabilities were calculated in Section 4.2) that the relationships are correct subject to the constraints.
• *Recompute clustering:* In a similar manner, if the two versions of the duplicate couple are in different clusters, then we add constraints to the hierarchical clustering algorithm and recompute the clustering for the affected matches.
• *Update lists:* We update all lists of ACs, e.g., moving entire sets of ACs of a match up or down one generation if we had originally placed the match in the wrong generation.
• *Modify past actions:* We reconsider all past ascents and descents related to all portions of the affected family tree. In particular, we may need to ascend or descend additional generations if we now believe that certain matches were originally placed in the wrong generation and we did not ascend from them for a sufficient number of generations.
• *Cumulative recalibration:* Our final calculation allows us to accommodate endogamy, where we might be consistently overestimating the proximity of the relationship between a match and the unsub, and hence underestimating the number of generations to ascend from a match; this calculation also mitigates the effects in cases where we misestimate the year of birth of the unsub. Whenever we find a duplicate couple, we keep a running average of the difference between the number of generations we originally ascended a primary match to get to its MRCA generation with the unsub, and the correct number of generations that we should have ascended. Then we add this running average (which can be positive or negative, and is rounded off to the nearest integer) to the number of generations that we ascend for all future primary matches.

### 7.2 Addressing the uncertainty in clustering

The hierarchical clustering results from Section 4.1 are accurate for many matches in generations 2 and 3 (as in, e.g., [13], [14], [15]), although our clustering algorithm was not reliable for matches that did not appear to be related to any other matches according to the autosomal matrix. Moreover, the clustering results degraded significantly at higher generations. In this subsection, we develop a unified approach to assess descending and ascending actions in the face of unreliable clustering. These inaccuracies are not due to the clustering algorithm itself, but rather are inherent in the data.

To deal with these inaccuracies, we now develop a method to characterize each clustering decision (each allocation of a match to an AC in each generation) as being either reliable or unreliable. Whereas in Section 6.2 we computed the exact generation of a connecting couple based on the clustering results of the matches that produced the ACs from which we were dual-descending, we now have five possible outcomes for the generation of a connecting couple: 1, 2, greater than 0, greater than 1, or greater than 2, where the inequalities stem from unreliable clustering results. In the latter three of these five cases, we can compute the probability distribution of the generation of the connecting couple, which allows us to incorporate this uncertainty into the benefit-to-cost ratio of a dual descent and of an ascending action.

To incentivize the use of reliable clustering information, when the current lowest-generation connecting couple is in generation greater than 3, we prioritize the investigation of matches that lead to a dual descent in which the generation of the new connecting couple is greater than 2, followed by greater than 1, followed by greater than 0, followed by 2, followed by 1. Similarly, when the current lowest-generation connecting couple is in generation 3, we prioritize the investigation of matches that lead to a dual descent in which the generation of the new connecting couple is 2, followed by 1, followed by greater than 0. Finally, when the current lowest-generation connecting couple is in generation 2, we prioritize the investigation of matches that lead to a dual descent in which the generation of the new connecting couple is 1, followed by greater than 0.

## 8 Insights for FIGG genealogists

In this section, we collect the insights that can be gleaned from our construction and use of FIGGsolver.

• *Keep a list for each of the unsub’s ACs:* For each of the unsub’s ACs, keep a list of couples in your family tree who could be this AC (i.e., could be an MRCA couple between a match and the unsub). This systematic approach allows the genealogist to stay focused on the most important information. Develop some understanding of the probability that each list currently contains the unsub’s AC (see §1.3 of the SI of [3] for details).
• *The year of birth of a match is helpful:* Updating the relationship of a match to the unsub after identifying a match (and hence learning his or her year of birth) can reduce the uncertainty in the number of generations to ascend the match in order to reach the MRCA generation between the match and the unsub.
• *Do not over-ascend:* Even in cases where it is easy to ascend many generations, only ascend a match to the generation of the MRCA couple between the match and the unsub.
• *Higher-cM matches are typically better:* All else being equal, it is preferable to investigate a match that has a higher shared cM with the unsub: it tends to lead to fewer generations of ascent to reach the generation of the MRCA couple between the match and the unsub (and hence to a smaller list of the unsub’s ACs), and to fewer generations of subsequent descent. While this is generally true, it is far from the whole story.
• *The age of a match is a double-edged sword:* We need to choose which match to investigate prior to knowing the match’s year of birth, which is revealed via a successful identification. Younger matches (e.g., those residing one or two generations below the unsub) typically require more generations of ascent to reach the MRCA generation between the match and the unsub (although sometimes it is easier for the genealogist to ascend in the more recent past), but the MRCA generation tends to be lower, which requires fewer generations of subsequent descent. In contrast, older matches (e.g., those residing one or two generations above the unsub) require fewer generations of ascent to reach the MRCA generation between the match and the unsub (which might be of the utmost importance in cases where ascents are difficult), but the MRCA generation tends to be higher, which requires more generations of subsequent descent.
• *A key driver of FIGGsolver’s results is the decreasing fertility rate over time:* In most cases, the overwhelming portion of the family tree is generated via descents, not ascents. This is largely due to the decreasing fertility rate throughout the last few centuries. Hence, it is imperative to make the lists of possible ACs of the unsub as short as possible, and with as high a probability as possible of containing the unsub’s AC.
• *The attractiveness of good supporting matches:* The key to making short lists of possible ACs of the unsub is community ascent: a high-cM primary match in isolation may not be as attractive as a lower-cM primary match that has good supporting secondary matches (e.g., matches that are more closely related to the primary match than the primary match is to the unsub). Moreover, a more distant supporting match can be more valuable than a closer supporting match; e.g., a secondary match that is the primary match’s parents only cuts the list size in half, while a secondary match that has an MRCA generation with the primary match that coincides with the MRCA generation between the primary match and the unsub can reduce the list size to one.
• *Consider ascending an additional generation after a failed dual ascent:* Due to the irreducible uncertainty in the relationship of each match to the unsub, if a dual ascent (e.g., of a primary match and a secondary match) does not result in an anticipated duplicate AC (i.e., the MRCA couple between these two matches), then consider ascending an additional generation from each match.
• *Sometimes it is worthwhile investigating matches whose MRCA generation with the primary match is at a higher generation than the MRCA generation of the primary match with the unsub:* This is often true when the primary match has a high shared cM with the unsub, but there are no secondary matches who are more closely related to the primary match than the primary match is to the unsub. After this new ascent, there will be a list of ACs in the lower generation that may contain the unsub’s AC, and a new list of ACs in the higher generation. In such cases (and with partial dual descents in Section 6.3), to find the unsub’s AC, it is often more efficient to ascend from the lower-generation list than to descend from the higher-generation list.
• *Hierarchical clustering allows you to ignore related matches after you find an MRCA couple:* If you already found an MRCA couple between a match and the unsub (and hence found an AC of the unsub), it is no longer necessary to investigate related matches that would lead to this same AC of the unsub. It is time to move on to matches in another cluster (i.e., matches that lead to a different AC of the unsub).
• *Dual descents help find connecting couples:* After generating two lists of ACs that have been made as small as possible via community ascents, the next step is typically to perform a dual descent from these two lists, with the aim of finding a connecting couple.
• *When deciding which match to investigate, keep in mind the subsequent descent:* You want to take ascending actions that eventually (i.e., after a subsequent dual descent) lead to finding a connecting couple, while adding the least number of people to the family tree.
• *In a dual descent, anticipate where the connecting couple resides:* Hierarchical clustering results can be used to compute the generation where the connecting couple resides. However, inaccuracies in the hierarchical clustering results can make this difficult.
• *It is efficient to find connecting couples incrementally:* For example, if you just found a connecting couple in generation 4, then half of the next dual descent should involve descending to the children of this connecting couple, with the goal of finding a new connecting couple in generation 3. However, inaccuracies in the hierarchical clustering results can make this incremental approach difficult to implement.
• *Consider an ascend–descend action after a failed dual descent:* If a dual descent from two lists does not result in an anticipated connecting couple, then it may be because at least one of these two lists contains ACs that are in a generation below the true generation of the MRCA couple between the match that generated these ACs and the unsub. In this case, the genealogist should consider an ascend–descend action, where one of the lists is ascended an additional generation, and then the recently-identified parents of the ACs in the list are descended.
• *Keep track of the age of female descendants to prevent over- and under-descending:* If you are aiming to descend to a particular generation, stop descending from females who could not have had children in that generation, and continue descending from females who could have had children in that generation.
• *Use inconsistent duplicate ACs or connecting couples as an opportunity to update:* While some of these updates are difficult without a computational algorithm, when duplicate ACs or connecting couples are found in unexpected generations or clusters, use these inconsistencies to update the probability distribution of the relationship between each match and the unsub, the lists, and the clustering results. Then review all past actions to determine whether additional generations of ascent or descent are required. Finally, use these inconsistencies to keep a running tab on how much you have been overestimating or underestimating the number of generations to ascend from matches, and recalibrate accordingly; this can mitigate the impact of endogamy and instances in which the year of birth of the unsub is unknown.
• *Understand the limitations of clustering results:* The hierarchical clustering results are inherently unreliable for generations above the generation of the great-grandparents. Try to understand these limitations, and the reliability of your knowledge about the generation of a connecting couple during a dual descent, and consider prioritizing actions that use reliable clustering information.

## CRediT authorship contribution statement

**Lawrence M. Wein:** Writing – original draft, Methodology, Formal analysis, Conceptualization. **Malo Sommers:** Writing – review & editing, Software.

## Declaration of competing interest

The authors declare that they have no known competing financial interests or personal relationships that could have appeared to influence the work reported in this paper.
